# A moderate 500-m treadmill walk for estimating peak oxygen uptake in men with NYHA class I-II heart failure and reduced left ventricular ejection fraction

**DOI:** 10.1186/s12872-018-0801-9

**Published:** 2018-04-16

**Authors:** Gianni Mazzoni, Biagio Sassone, Giovanni Pasanisi, Jonathan Myers, Simona Mandini, Stefano Volpato, Francesco Conconi, Giorgio Chiaranda, Giovanni Grazzi

**Affiliations:** 1 Department of Biomedical and Specialty Surgical Sciences, University of Ferrara, Ferrara, Italy; 2 Public Health Department, AUSL Ferrara, Ferrara, Italy; 3 Department of Medicine, Division of Cardiology, Cento Hospital, AUSL Ferrara, Ferrara, Italy; 4 Department of Medicine, Division of Cardiology, “Delta” Hospital, AUSL Ferrara, Ferrara, Italy; 5 Veterans Affairs Palo Alto Health Care System, Palo Alto, CA USA; 6 Stanford University School of Medicine, Stanford, CA USA; 7 Center of Biomedical Studies Applied to Sport, University of Ferrara, via Gramicia 35 -, 44123 Ferrara, Italy; 8 Department of Medical Sciences, University of Ferrara, Ferrara, Italy; 9 General Directorship for Public Health and Integration Policy, Emilia-Romagna Region, Bologna, Italy

**Keywords:** Heart failure, Left ventricular dysfunction, Cardiorespiratory fitness, Walking test

## Abstract

**Background:**

Maximal cardiopulmonary exercise testing (CPX) is the gold-standard for cardiorespiratory fitness assessment in chronic heart failure (CHF) patients. However, high costs, required medical supervision, and safety concerns make maximal exercise testing impractical for evaluating mobility-impaired adults. Thus, several submaximal walking protocols have been developed and currently used to estimate peak oxygen consumption (VO_2_peak) in CHF patients. However, these tests have to be performed at close to maximum exercise intensity. The aim of this study was to examine the validity of a 500-m treadmill-walking test carried out at moderate intensity for estimating VO_2_peak in community-dwelling adult and elderly patients with CHF and reduced left ventricular ejection fraction (HFrEF).

**Methods:**

Forty-three clinically stable men with HFrEF (age 67.7 ± 9.2 years, and left ventricular ejection fraction, LVEF 38% ± 6%) underwent exercise testing during an outpatient cardiac rehabilitation/secondary prevention program. Each patients completed a CPX, and a moderate and self-paced (11–13/20 on the Borg scale) 500-m treadmill-walking test. Age, weight, height, walk time, and heart rate during the 500-m test were entered into prediction equations previously validated for VO_2_peak estimation from a 1000-m walking test in patients with cardiovascular disease and preserved LVEF.

**Results:**

Directly measured and estimated VO_2_peak values were not different (21.6 ± 4.9 vs 21.7 ± 4.6 mL/kg/min). The comparison between measured and estimated VO_2_peak values yielded a correlation of *R* = 0.97 (SEE = 0.7 mL/kg/min, *P* < 0.0001). The slope and the intercept coincided with the line of identity (Passing and Bablock analysis, *P* = 0.50). Residuals were normally distributed, and the examination of the Bland-Altman analysis do not show systematic or proportional error.

**Conclusions:**

A moderate and self-regulated 500-m treadmill-walking test is a valid tool for VO_2_peak estimation in patients with HFrEF. These findings may have practical implications in the context of transitioning from clinically based programs to fitness facilities or self-guided exercise programs in adults and elderly men with HFrEF.

**Electronic supplementary material:**

The online version of this article (10.1186/s12872-018-0801-9) contains supplementary material, which is available to authorized users.

## Background

Chronic heart failure (CHF) is an increasingly important health problem because of the aging population, improved survival rate after acute cardiovascular events, and the escalating costs attributable to symptoms and associated repeated hospitalizations, despite optimal medical therapy [1]. Cardiorespiratory fitness (CRF), usually best reflected by peak exercise oxygen consumption (VO_2_peak), has been shown to be a powerful and independent prognostic marker in patients with CHF [2, 3], and is strongly related to walking speed [4, 5].

Walking tests of varying distance and times are commonly used to assess exercise tolerance in various clinical conditions (including CHF). These tests have to be performed at an intensity close to maximum. In fact, the participants are instructed to “*cover as much ground as you possibly can*” during a certain time [6] or “*to walk as fast as possible*” for a certain distance [7, 8].

However, daily activities rarely require maximal effort, and thus the ability to perform sustained submaximal exercise is an important component of health-related fitness assessment. In this respect, examination of submaximal exercise capacity can be useful not only to functionally evaluate patients but also for developing appropriate exercise prescriptions, adjusting the medical regimen, and identifying the need for further diagnostic interventions [9].

Submaximal walking tests for CRF assessment may be useful for measuring improvements not captured by VO_2_peak such as the capacity to perform activities of daily living, particularly for populations whose health limits their ability to exercise at maximal effort [10]. Slow walking in older adults reflects disease severity and underlying frailty, and has significant consequences for the individual, and the public health system [11]. In addition, the need of using simple exercise tests to improve the admission on cardiac rehabilitation/secondary prevention programs has been recently emphasized, particularly for low-resource settings [12].

The moderate speed maintained during a 1-km treadmill walk (1 k-TWT) has been demonstrated to be a valid and simple tool for CRF estimation [13, 14], and is inversely related to survival [15, 16], and hospitalization [17] in outpatients with cardiovascular disease (CVD) and preserved left ventricular ejection fraction (LVEF). However, the 1 k-TWT can be time-consuming, particularly when performed by functionally limited patients. A shorter test providing similar functional information while limiting physical demands of patients and taking less time could be valuable. Thus, the aim of this study was to examine the validity of a 500-m treadmill-walking test carried out at moderate intensity for estimating peak oxygen consumption (VO_2_peak) in community-dwelling adult and elderly patients with CHF and reduced left ventricular ejection fraction (HFrEF).

## Methods

### Participants

43 consecutive medically stable male outpatients with HFrEF (LVEF ≤45%), aged 35 to 83 yrs. (67% over 65 yrs), classified as NYHA class I-II are included in the study. Each subject completed a clinical evaluation including personal and family history and a medical examination. Left ventricular ejection fraction was derived from recent echocardiographic evaluation. Standard blood chemistry analyses previously performed were recorded. Weight and height were measured and used to calculate body mass index (BMI). Blood pressure (BP) was measured, and hypertension was defined as systolic BP ≥ 140 mmHg, diastolic BP ≥ 90 mmHg, or use of antihypertensive agents. All patients performed a maximal cardiopulmonary exercise test (CPX) for direct VO_2_peak determination, and a 500-m moderate treadmill-walking test for VO_2_peak estimation. Patients were instructed not to change dietary habits, not to consume any food or beverages except water for ≥2 h before testing, and not to engage in any type of physical activity for two days before testing. All were evaluated while receiving their usual medications and were on a stable medical regimen for at least three months before testing. 37 (86%) subjects were receiving β-blockers as follows: bisoprolol (*n* = 19, 6 ± 3 mg/d), carvedilol (*n* = 11, 31 ± 19 mg/d), and metoprolol (*n* = 7, 135 ± 42 mg/d).

### Exercise testing procedures

#### Cardiopulmonary exercise testing

VO_2_peak was determined using a treadmill ramp protocol beginning at speed of 1.5 mph (2.4 km/h) and 1.5% grade with subsequent increments of 0.1 mph and 0.5% grade every thirty seconds [18]. Patients performed the maximal test until subjective exhaustion, exertional chest pain or other untoward findings that would necessitate termination. Exercise was considered adequate if it was limited by dyspnea or muscle fatigue (Rate of perceived exertion, RPE ≥ 18/20), and by the attainment of at least two of the three following criteria: heart rate value ≥85% of the age-predicted maximum, VO_2_ plateau approaching maximal exertion, and a respiratory exchange ratio ≥ 1.05 [19, 20]. Tight gripping of the handrails was not permitted; finger or palm placement on the handrails was allowed for balance only when necessary. Standard 12-lead electrocardiograms were continuously monitored and recorded during the test (Quark T12x, Cosmed, Rome, Italy). Gas-exchange measurements were performed using a metabolic cart (Omnia 1.5, Cosmed, Rome, Italy). Calibration of the system was performed before each test using a three-liter syringe to calibrate the flowmeter and by using gases with known oxygen, carbon dioxide and nitrogen concentrations to calibrate the gas analyzers. VO_2_ and carbon dioxide output were acquired breath-by-breath and averaged over 15 s intervals. VO_2_peak was defined as the highest level of VO_2_ achieved during the test.

#### 500-m treadmill-walking test

Within one week from the CPX, each patient performed a 500-m treadmill-walking test. Each patients were informed to adopt a comfortable walking pace sustainable for 10 to 20 min. Participants were educated to maintain a moderate perceived exercise intensity using the Borg 6–20 scale. The test was performed on the level, and began with a preliminary phase at 2.0 km/h, with subsequent increases of 0.3 km/h every 30 s up to a walking speed corresponding to a perceived exertion of 11–13 on the Borg scale. The 500-m walk was then started and the rate of perceived exertion acquired every 2 min. Walking speed was adjusted by the operator following the patient’s perceived intensity. In this way, the exercise intensity was individualized and maintained at moderate perceived exertion.

Heart rate was monitored continuously during the test using a Polar RS100 heart rate monitor (Polar Electro, Kempele, Finland). Blood pressure was monitored before and immediately after the test. Heart rate was averaged every five seconds and mean and maximal values during the test were determined. Age, height, weight, time to walk 500-m multiplied by two, and heart rate were entered into the equations developed to estimate VO_2_peak by the 1 k-TWT [13]. The two equations were determined using a multivariate forward stepwise regression procedure [13]. A coefficient of determination was calculated for each variable for the VO_2_peak estimation. After removal of variables that were not significant, the model included age, BMI, walking speed and heart rate. The model was set as follows:

$$ \mathrm{Y}={\upbeta}_0\hbox{--} {\upbeta}_1{\mathrm{X}}_1\hbox{--} {\upbeta}_2{\mathrm{X}}_2\hbox{--} {\upbeta}_3{\mathrm{X}}_3\hbox{--} {\upbeta}_4{\mathrm{X}}_4 $$

where Y = directly measured VO_2_peak; β = regression coefficient for each of the independent variables; X_1_ = mean walking speed in km/h; X_2_ = BMI in weight/height^2^; X_3_ = age in years; and X_4_ = higher heart rate in beats per minute (bpm). The resulting predictive equations were:

$$ \left[33.42+2.79\ \left(\mathrm{walking}\ \mathrm{speed}\right)\hbox{--} 0.49\ \left(\mathrm{BMI}\right)\hbox{--} 0.14\ \left(\mathrm{age}\right)\right] $$

and

$$ \left[46.11+4.41\ \left(\mathrm{walking}\ \mathrm{speed}\right)\hbox{--} 0.40\ \left(\mathrm{BMI}\right)\hbox{--} 0.30\ \left(\mathrm{age}\right)\hbox{--} 0.11\ \left(\mathrm{heart}\ \mathrm{rate}\right)\right] $$

for patients taking and not taking β-blockers, respectively.

#### Data analysis

Normal distribution of collected data has been verified by using D’Agostino Pearson test. Therefore, the predicting equations previously developed and validated [13] were applied using Pearson product moment correlations, SEE and paired t-test for comparison of measured and predicted VO_2_peak. Passing and Bablok regression analysis was used to determine the relationship between measured and predicted values. Agreement between methods has been assessed calculating the Concordance Correlation Coefficient. Appropriateness of the model was assessed using Bland-Altman analysis and normal probability plots of the residuals. The level of statistical significance was set at *P* < 0.05. Statistical analyses were performed using the package Medcalc 16.2 software (Ostende, Belgium).

## Results

Both the CPX and the 500-m treadmill-walking tests were completed by all subjects without complications. Three subjects did not satisfy the criteria for adequate effort. One patient interrupted the test prematurely because of mask intolerance (i.e. claustrophobic). The analysis thus included the results of 39 subjects. Descriptive characteristics of the study population are presented in Table 1. CPX test results are presented in Additional file 1: Table S1.

**Table 1:** Descriptive characteristics of the participants

n	39
Age (y)	67.7 (9.2)
Body mass index (kg/m^2^)	28.8 (3.8)
Left ventricular ejection fraction (%)	38 (6)
Ischemic etiology (%)	72
NYHA functional class I/II (%)	62/38
Serum sodium (mEq/L)	140 (2)
Cardiovascular risk factors	
Family history of CVD (%)	66
Hypertension (%)	72
Fasting glucose (mg/dl)	105 (25)
Total cholesterol (mg/dl)	167 (47)
HDL cholesterol (mg/dl)	42 (9)
Triglycerides (mg/dl)	107 (52)
Serum creatinine (mg/dl)	1.03 (0.2)
Current smoking (%)	6
Medical history (%)	
Coronary artery by-pass graft	41
Myocardial infarction	39
PTCA	30
Valvular repair/replacement	11
Other	8
Medications (%)	
ACE inhibitor or ARB	89
Aspirin	80
β-blockers	89
Calcium antagonists	14
Diuretics	81
Statins	75

Data are presented as mean (standard deviation) or percentage. *ACE* angiotensin-converting enzyme, *ARB* angiotensin receptor blocker, *CVD* cardiovascular disease, *NYHA* New York Heart Association, *PTCA* Percutaneous Transluminal Coronary Angioplasty

Average walking speed during the 500-m treadmill-walking test was 4.37 ± 1.08 km/h. Mean heart rate was 90 ± 21 bpm, representing 59% ± 14% of the age-predicted maximal heart rate (based on 220-age).

VO_2_peak values measured by CPX and predicted from the 500-m treadmill-walking test resulted 21.6 ± 4.9 mL/kg/min and 21.7 ± 4.6 mL/kg/min respectively (*t* = 0.9, *P* = 0.37). 500-m test results are presented in Table 2.

**Table 2:** 500-m treadmill walking test results

Variable	Mean (SD)
Exercise time (min:sec)	6:51 (2:24)
Mean walking speed (km/h)	4.37 (1.08)
Highest walking speed (km/h)	4.54 (1.06)
Mean heart rate (bpm)	90 (21)
Highest heart rate (bpm)	94 (17)
Estimated VO_2_peak (mL/kg/min)	21.7 (4.7)

Data are presented as mean (standard deviation)

**Fig. 1:**
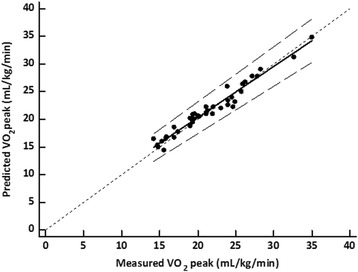
Regression of the VO_2peak_ estimated from 500-m moderate treadmill-walking test on the measured VO_2peak_ (y = 2,37 + 0,90×). The diagonal line represents the line of perfect agreement (line of identity), and the dotted lines represent confidence interval lines

**Fig. 2:**
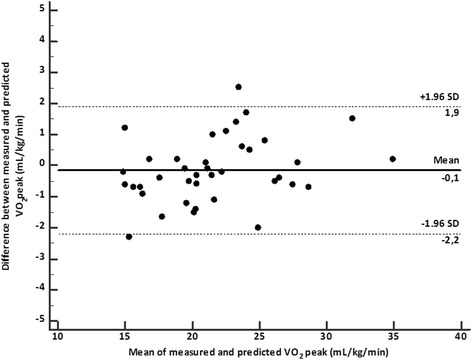
Difference compared to the mean of VO_2_ measured by CPX and estimated from 500-m walk (Bland and Altman plot)

The correlation coefficient between measured and predicted VO_2_peak was 0.97 (*P* < 0.0001), and the SEE was 0.7 mL/kg/min. The slope and the intercept of the relationship between measured and predicted VO_2_peak were not significantly different from the line of identity (Passing and Bablock analysis, *P* = 0.50, Fig. 1). The Concordance Correlation Coefficient resulted 0.97. Residuals were normally distributed with a mean residual value of − 0.1 mL/kg/min. Examination of the Bland-Altman analysis do not show systematic or proportional error (Fig. 2).

## Discussion

The major finding of this study was the VO_2_peak prediction by a 500-m treadmill-walking test carried out at moderate intensity in subjects with HFrEF. Predicted VO_2_peak strongly paralleled VO_2_peak measured directly by CPX. This was supported by the high correlation and concordance coefficients, and by the small SEE (0.7 mL/kg/min). These findings suggest that the equations validated for predicting VO_2_peak using a 1000-m moderate treadmill walk in outpatients with CVD and preserved ejection fraction, are similarly appropriate for predicting VO_2_peak when using a 500-m moderate treadmill walk in CHF outpatients with mild to moderately reduced ejection fraction.

These results are in agreement with those obtained by others that have attempted to predict VO_2_peak using sub-maximal walking protocols, mainly in healthy subjects and in patients with CVD and preserved LVEF [5, 7, 21–24].

In our study, by using population specific-equations developed for men with CVD and preserved LVEF, VO_2_peak was accurately estimated in adults and elderly subjects with HFrEF by performing a 500-m moderate walk. In addition, the relationship between moderate and perceptually regulated walking and cardiorespiratory fitness in CHF outpatients has not been determined in previous studies. Correlation coefficients observed in our original study [13] and the present results further support the strong association between walking speed and peak VO_2_ [4].

The 500-m treadmill-walking test relies on the familiar task of moderate walking, which is the most common activity engaged in by adults. In particular, older adults reported that walking was their preferred form of exercise [25, 26]. The habitual nature of walking reduces the possibility that lack of familiarity with the task reduces the predictive accuracy of the results. This characteristic may also make the 500-m walking test particularly appropriate for less fit patients or for those whom walking is their preferred form of physical activity. This reduces the influence that lack of familiarity with the task might have on the accuracy of the test. Various walking tests have also been proposed as functional tools s for patients with CHF; thus, we felt that a submaximal test such as the 500-m treadmill-walking test would be valuable in this population, particularly for low-resource settings [12].

An advantage of the 500-m treadmill-walking test is the fact that it is performed at an individualized and patient-determined moderate intensity (11 to 13 on the RPE scale). This was confirmed by the average 59% of the age-predicted maximal heart rate value in the present study, which falls within current recommended limits (55% to 69%) for moderate intensity [27]. Moreover, the average heart rate during the 500-m test was close and well correlated with the heart rate value at ventilatory threshold during CPX (90 ± 21 bpm vs 93 ± 16 bpm, *R* = 0.83, *P* < 0.0001).

As such, the test may also serve as a learning trial for proper intensity for an exercise prescription. In fact, the exercise intensity at an RPE value between 11 and 13/20 has been associated with the lactate threshold, independent of training state [28]. Aerobic conditioning at such an intensity has been demonstrated to be safe and optimal to enhance cardiorespiratory function in patients with chronic disease [28].

The 500-m treadmill-walking test is a simple test that could be applied to stable mild-to-moderate CHF subjects, accurately reflecting activities of daily living. The current results provide insights into the underlying characteristics and treatment of CHF patients with reduced left ventricular ejection fraction, which may be useful to help quantify CRF improvement or deterioration.

### Study limitations

First, the small number of patients is a limit of this study. Second, our study comprised male participants only: thus, the results may not be generalizable to women. Third, participants were in the mid-range of reduced LVEF, and therefore the results may not apply to patients with more impaired ventricular function. Fourth, these results were obtained from patients with an interest in participating in an exercise-based secondary prevention program. Finally, reproducibility was not assessed in this study. However, we previously demonstrated good cross-validation and reproducibility among cardiac outpatients performing the test over the 1000-m distance [13]. Therefore, external validation of our findings is needed. In particular, future work should focus on the application of the protocol to women with CVD and to subjects with more impaired CRF, as well as to examine the prognostic value of the 500-m moderate walking test, including the determination of clinically meaningful cut-points.

## Conclusions

In outpatients with mild-to-moderate CHF, the determination of submaximal exercise capacity by a moderate and perceptually-regulated the 500-m treadmill-walking test, accurately predicts VO_2_peak. Since it is important to document functional capacity in heart failure patients over time, the 500-m treadmill-walking test represents a simple and potentially useful tool in the serial evaluation of clinical status or the response to therapeutic interventions including exercise prescription. While the CPX remains the gold-standard for cardiorespiratory fitness assessment in chronic heart failure patients, the 500-m walking test may also provide an inexpensive screening tool in this and other patient groups in which it has been validated.

## Additional file

Additional file 1: **Table S1.** Cardiopulmonary exercise test results. (DOCX 16 kb)

## Abbreviations

1 k-TWT: 1-km treadmill walking test
BMI: body mass index
BP: blood pressure
CHF: chronic heart failure
CPX: Cardiopulmonary exercise testing
CRF: cardiorespiratory fitness
CVD: cardiovascular disease
HFrEF: heart failure and reduced left ventricular dysfunction
LVEF: left ventricular dysfunction
NYHA: New York Hear Association
RPE: rate of perceived exertion
SEE: standard errors of estimate
VO_2_peak: peak oxygen uptake
VT: Ventilatory threshold
