# Supplementary material for: A moderate 500-m treadmill walk for estimating peak oxygen uptake in men with NYHA class I-II heart failure and reduced left ventricular ejection fraction
Source: BMC Cardiovasc Disord. 2018 Apr 16;18:67. doi: 10.1186/s12872-018-0801-9 (PMC5902976; doi:10.1186/s12872-018-0801-9)
Supplement: Supplementary file 1 — Table S1. Cardiopulmonary exercise test results. (DOCX 16 kb) [file 12872_2018_801_MOESM1_ESM.docx]

Table S1. Cardiopulmonary exercise test results.

|  | **Rest** | **VT** | **RCP** | **Maximum** | |
| --- | --- | --- | --- | --- | --- |
|  |  |  |  | Achieved | pp (%) |
| **Power output** |  |  |  |  |  |
| Exercise time (min:sec) | 0:00 | 6:21 (2:27) | 8:44 (2:37) | 9:59 (2:49) | - |
| Speed (km/h) | 0.0 | 4.3 (0.8) | 5.0 (0.9) | 5.5 (0.9) | - |
| Grade (%) | 0.0 | 7.3 (2.5) | 9.7 (2.6) | 11.2 (3.1) | - |
| **Metabolic** |  |  |  |  |  |
| VO_2_ (mL/kg/min) | 6.3 (0.9) | 16.6 (3.4) | 19.3 (4.0) | 21.7 (5.2) | 89 (15) |
| Absolute VO_2_ (L/min) | 0.54 (0.08) | 1.441 (0.307) | 1.666 (0.372) | 1.870 (0.449) | 89 (15) |
| METs | 1.8 (0.3) | 4.7 (1.0) | 5.5 (1.2) | 6.2 (1.5) | 89 (15) |
| VCO_2_ (L/min) | 0.44 (0.08) | 1.206 (0.336) | 1.621 (0.408) | 2.039 (0.571) | - |
| Respiratory exchange ratio (VCO_2_/ VO_2_) | 0.79 (0.07) | 0.83 (0.09) | 0.97 (0.08) | 1.08 (0.08) | - |
| **Cardiovascular** |  |  |  |  |  |
| Heart rate (bpm) | 56 (12) | 93 (16) | 104 (20) | 122 (24) | 80 (14) |
| Oxygen pulse (VO_2_/HR, mL/bpm) | 9.9 (2.2) | 15.7 (3.5) | 16.0 (3.5) | 15.5 (3.4) | 124 (34) |
| Heart rate reserve (peak-rest, bpm) | 66 (12) | | | | |
| Heart rate decrease at 1 min (beats) | 21 (10) | | | | |
| Systolic blood pressure (mm Hg) | 120 (11) | - | - | 168 (18) | - |
| Diastolic blood pressure (mm Hg) | 75 (7) | - | - | 72 (9) | - |
| **Ventilatory** |  |  |  |  |  |
| FEV_1_ (L/min) | 2.70 (0.68) | - | - | - | 83 (17) |
| Ventilation (VE, L/min) | 17 (3) | 40 (10) | 53 (12) | 75 (19) | - |
| MVV (L/min) | - | - | - | 109 (27) | - |
| VE/MVV | - | - | - | 0.72 (0.16) | - |
| Respiratory rate (br/min) | 19 (4) | 23 (5) | 26 (4) | 36 (6) | - |
| VE/VO_2_ (L/min/L/min) | 31 (6) | 28 (5) | 32 (5) | 41 (10) | - |
| VE/VCO_2_ (L/min/L/min) | 38 (9) | 33 (5) | 33 (5) | 38 (8) | - |
| VE/VCO_2_ slope | - | 30.8 (5.6) | 33.2 (6.5) | 36.4 (8.7) | 117 (29) |
| PetCO_2_ (mm Hg) | 31 (4) | 37 (5) | 36 (5) | 32 (7) | - |
| Oxygen uptake efficiency slope | - | - | - | 2255 (406) | 122 (30) |
| Exercise oscillatory ventilation (n/%) | 7/19 | | | | |

Data are presented as mean (standard deviation). FEV_1_, forced expiratory volume in 1 s; METs, metabolic equivalents; MVV, maximal voluntary ventilation; pp, percent predicted; PetCO_2_, end tidal carbon dioxide pressure; RCP, respiratory compensation point; VE, ventilation; VCO_2_, carbon dioxide output; VO_2_, oxygen uptake; VT, ventilatory threshold.
